# Effectiveness of combination the photobiomodulation therapy and triamcinolone acetonide on managing oral major aphthous ulcer in Crohn’s disease: A case report

**DOI:** 10.1097/MD.0000000000043336

**Published:** 2025-07-18

**Authors:** Shahed Kuraitby, Alaa ALhomsi, Abeer A. Aljoujou

**Affiliations:** a Department of Oral Medicine, Faculty of Dental Medicine, Damascus University, Damascus, Syria.

**Keywords:** case report, Crohn’s disease, major aphthous ulcer, photobiomodulation therapy, triamcinolone acetonide

## Abstract

**Rationale:**

Inflammatory bowel diseases like Crohn’s disease can manifest with oral ulcers that negatively impact quality of life. Managing these stubborn symptomatic lesions requires an integrated medical and dental approach.

**Patient concerns:**

A 23-year-old male was diagnosed with Crohn’s disease 3 years ago. He was referred to the oral medicine department due to a painful 3 cm ulcer on the dorsum of the tip of his tongue, which was interfering with his ability to eat and drink.

**Diagnoses:**

A gastrointestinal endoscopy revealed a rectal ulcer, a cecal ulcer, superficial erosion, and scattered millimetric warts in the rectal area.

**Interventions:**

The patient was treated with a daily dose of azathioprine (50 mg) for 2 years. Once the clinical symptoms improved, the medication was discontinued. The major aphthous ulcer was managed with a combined treatment of 0.1% triamcinolone acetonide oral base and photobiomodulation laser therapy.

**Outcomes:**

This dual therapy approach aimed to reduce pain, accelerate healing, and prevent scarring.

**Lessons:**

Collaborative care between gastroenterology and dentistry led to better symptom control and clinical outcomes for this patient with miserable mouth ulcers from Crohn’s disease. The case highlights the benefits of a transdisciplinary treatment strategy targeting both oral and intestinal manifestations of inflammatory bowel disorders.

## 1. Introduction

Inflammatory bowel disease (IBD) is a chronic, relapsing condition that affects the immune system of the gut and includes ulcerative colitis and Crohn’s disease (CD).^[1]^ The exact cause of CD is unknown, but environmental, immunological, and genetic factors can increase the likelihood of developing and worsening the disease.^[2]^ Extraintestinal manifestations (EIMs) may occur in 21%–47% of patients with CD and can significantly impact a patient’s quality of life (QoL).^[3]^ EIMs can affect various body systems, including the musculoskeletal system, eyes, skin, and oral cavity.^[4]^ Patients with CD often experience oral manifestations, which are observed in a range of 5%–60% of cases, with higher incidence rates in males and children.^[5]^ Interestingly, these oral symptoms may appear several months or even years before any intestinal symptoms become apparent. Oral symptoms of CD can be classified as either specific or nonspecific, with nonspecific ulcers being the more prevalent type.^[6]^ The exact mechanisms causing oral and dental issues in CD are not yet fully understood. However, several underlying pathological factors have been proposed to explain the development of oral manifestations in Crohn’s. For instance, active CD-related oral EIM might arise from changes induced by cytokines in the gastrointestinal tract, which also affect the oral cavity.^[6]^ Individuals with these ulcers may experience substantial pain and discomfort.^[7]^ With a prevalence ranging from 0.7% to 20%, aphthous ulcers are the most common type of oral EIM.^[8]^ The characteristic feature is the presence of oral shallow, circular ulcers on the buccal and labial mucosa, surrounded by an erythematous halo. These ulcers may occur alone or in conjunction with recurrent aphthous stomatitis.^[9]^ In mild cases, topical treatments such as lidocaine, steroids, and nonsteroidal anti-inflammatory pastes are used to treat these ulcerations. However, in severe cases, systemic steroids and immunosuppressive drugs are required for treatment.^[5]^ Recent studies have shown that various lasers, such as Nd: YAG, CO_2_, and diode lasers, are more effective in reducing pain and hastening the healing process for aphthous ulcers.^[10]^

This case report aimed to demonstrate an effective treatment for major oral aphthous ulcers as extraintestinal manifestations of CD. The treatment plan combined triamcinolone acetonide 0.1% and photobiomodulation therapy (PBMT), with the goal of accelerating the treatment response, alleviating pain, and minimizing scarring. This work has been reported in line with the SCARE 2020 criteria.^[11]^

## 2. Case report

**Figure 1.**
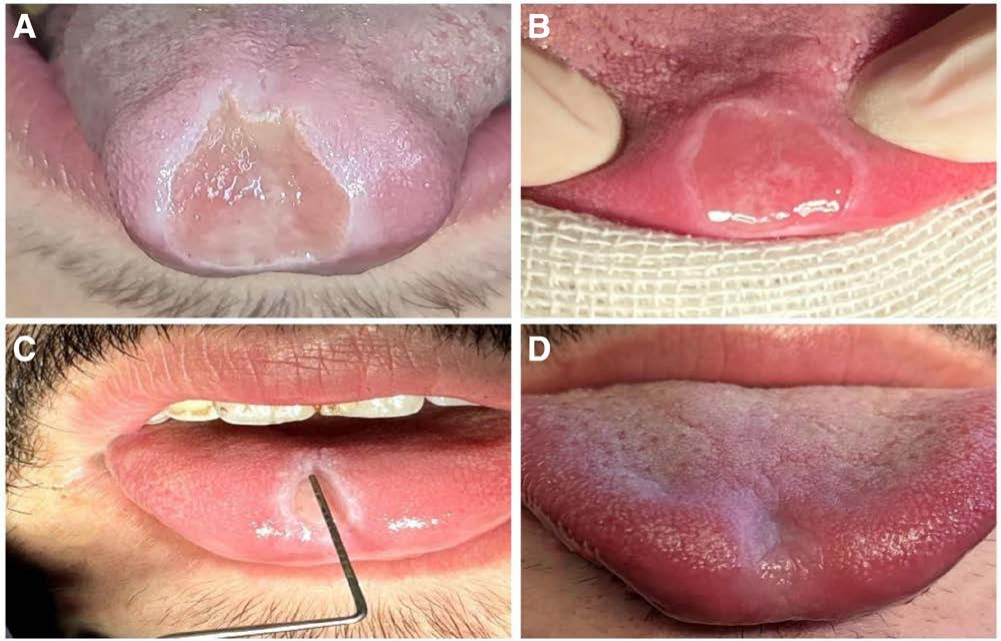
**The patient’s condition at each stage.** (A) Before the treatment, the initial diagnosis revealed a 3 cm aphthous ulcer on the tip dorsum of the tongue (B) the size of the aphthous ulcer on the seventh day of treatment. (C) The size of the aphthous ulcer on the eleventh day of the treatment (D) Ulcer healing after 16 days with the combined treatment of photobiomodulation therapy and triamcinolone acetonide oral base.

A 23-year-old male patient with a 3-year history of CD was referred to the Department of Oral Medicine at the Faculty of Dental Medicine, Damascus University. The patient presented with an extremely painful ulcer, 3 cm in diameter, on the tip dorsum of the tongue (Fig. 1). Due to its sensitive location, the ulcer interfered with eating, drinking, and speaking, leading to weight loss and a decreased QoL.

In his medical history, the patient stated that the ulcer had appeared 8 days earlier and had become bigger and more painful. Three years ago, he was diagnosed with CD after undergoing a diagnostic procedure that included a digestive endoscopy and biopsies from the rectal and ileal regions. During the colonoscopic examination, a rectal ulcer, superficial erosion, and several small rectal warts were observed. Additionally, extensive erosions were found in the cecum. Biopsies taken from both the rectal and cecal lesions revealed widespread ulceration, accompanied by acute and chronic inflammation as well as granulation tissue. The patient underwent immunological testing 3 years ago at the time of CD diagnosis, showing a positive ASCA and negative pANCA. The diagnosis was confirmed not solely based on these markers but through a comprehensive assessment, including endoscopic findings, histological biopsy, and initial clinical symptoms—chronic diarrhea, abdominal pain, and weight loss—present at that time. The diagnosis was confirmed, and he was treated with a daily dose of azathioprine (50 mg) for 2 years. Once the clinical symptoms improved, the medication was discontinued. No previous smoking history.

Intraoral examination revealed a round-shaped, painful ulcer with erythematous borders. The extraoral examination includes inspecting the area around the oral fossa, including the lymph nodes in the head and neck, major salivary glands, and cranial nerves. This examination did not reveal any abnormalities. Based on the presenting signs and symptoms, the patient was diagnosed with a major aphthous ulcer. Blood tests, including ferritin, folic acid, and serum vitamin B12 levels, were ordered to screen for nutritional deficits related to CD. All values were in the normal range (Table 1). The patient was referred to a gastrointestinal specialist to confirm the absence of active CD. The consultation revealed that the disease was under control.

**Table 1:** Blood test results for the patient.

Blood tests	Result	Reference range
White blood cells (WBC)	10 × 10^3^/mm^3^	4–11
Red blood cells (RBC)	5.55 × 10^6^/mm^3^	4.3–5.7 × 10^6^/mm^3^
Hemoglobin (HB)	15.1 g/dL	13.2–17.3 g/dL
Hematocrit (Hct)	47.00%	39–49%
Platelets (Plt)	354 × 10^3^/mm^3^	150–450 × 10^3^/mm^3^
Vitamin B_12_	891 pg/mL	180–900 pg/mL
Ferritin	84.3 ng/mL	24–336 ng/mL
Folic acid	3.9 ng/mL	3.7–16.1 ng/mL

We recorded the patients provoked pain (due to contact with the ulcer) using a 0–10-point visual analog scale, with a score of (10). We also used the oral health impact profile (OHIP)-14 questionnaire to measure the impact of oral disease on QoL. The OHIP-14 is a self-administered questionnaire that assesses QoL using 14 questions, which capture 7 dimensions: functional limitation, physical pain, psychological discomfort, physical disability, psychological disability, social disability, and handicap. Each dimension is measured by 2 questions (2 questions measure each dimension). Scores can range from 0 to 56 and are calculated by summing the ordinal values; higher OHIP-14 scores indicate worse QoL, and lower scores indicate better QoL. The Arabic version of the OHIP-14 was used.

The patient was informed about the treatment plan, and written consent was obtained before starting the treatment, which included triamcinolone acetonide 0.1% (KENALOG IN ORABASE, Unipharma, Damascus, Syria). This medication was topically applied to the aphthous ulcer 3 times a day for 10 days using a swab, up to 1 cm in size, ideally after eating and mouthwashing. The patient was instructed not to consume food or drink for at least an hour after the medication was applied. Triamcinolone acetonide 0.1% was combined with PBMT using a diode laser, administered 3 times a week for 2 weeks. During the initial visit, the laser treatment was promptly started with the parameters shown in Table 2. Six points were targeted according to lesion size.

**Table 2:** Details of laser device used in patient treatment.

Manufacturer	PIOON Company
Model identifier	MER-G10
Emitter type	(AsGaAI) arsenide gallium aluminum
Type of laser	Diode laser
Operation mode	Continuous
Wave length	650 nm
Power	100 mW
Exposure time	20 s
Delivery system	Photobiomodulation handpiece
Spot size	0.5 cm^2^
Energy density per point	3 J/cm^2^
Application technique	Contact mode

The patient’s pain was recorded daily using the visual analog scale, and it completely disappeared by the seventh day (Table 3). The size of the ulcer was recorded on the days that the patient came for laser application (Table 3), and it was fully healed within 16 days without any scarring (Fig. 1). The patient’s general health improved, particularly with regard to nutritional intake. These improvements are reflected in the OHIP-14 results, indicating an overall enhancement in his QoL (Table 3). The patient was followed up 1 month and 3 months after treatment, and no recurrence of the aphthous ulcer was observed.

**Table 3:** Pain reduction (VAS), the size of the lesion and OHIP-14 score during the treatment days.

VAS	Before the treatment	10
	First day	8
	Second day	6
	Third day	5
	Fourth day	4
	Fifth day	2
	Sixth day	1
	Seventh day	0
Size of the lesion	Before the treatment	3 cm
	First day	3 cm
	Third day	3 cm
	Seventh day	1.7 cm
	Ninth day	1 cm
	Eleventh day	7 mm
	Thirteenth day	3 mm
	Sixteenth day	Complete healing
OHIP-14	Before the treatment	51
	Third day	28
	Seventh day	0

OHIP = oral health impact profile, VAS = visual analog scale.

## 3. Discussion

CD is a chronic and relapsing IBD that has been increasingly prevalent over the past decade, creating a significant burden on healthcare systems.^[12,13]^ The etiology of CD remains complex; however, it is increasingly recognized that CD results from an inappropriate mucosal inflammatory response to intestinal bacteria in genetically susceptible individuals.^[14]^ The majority of CD symptoms affect the gastrointestinal system, although oral symptoms may also occur.^[15]^ These symptoms can develop over the course of the disease, appear before diagnosis, or result from dietary deficiencies associated with the condition.^[16]^ Earlier studies found no association between disease activity and oral lesions, suggesting that these lesions are less likely to be linked to active disease. However, up to 30% of affected patients may still experience oral lesions even after the disease is under control.^[5]^ Oral lesions can affect any part of the oral cavity, including the lips, tongue, hard and soft palate, salivary glands, gingiva, and teeth.^[16]^ Kalmar^[17]^ first reported oral lesions in 2 patients with CD in 1969. This was followed by Dudeney’s description of another CD patient with oral manifestations. Aphthous ulcers are common and frequent in these patients, and it is essential for healthcare professionals to differentiate between typical aphthous ulcers and those that are more severe, which may be linked to systemic disease.^[18]^ However, aphthous ulcers are frequently more severe and widespread in CD patients.^[19]^

Aphthous ulcers can be categorized as minor, major, or herpetiform according to their size, duration, and potential for scarring. Small ulcers usually heal without scarring, while large ulcers heal with scarring.^[16]^ There is growing discussion about the crucial role that immunopathological responses play in the pathophysiology of aphthous ulcers. One possible explanation for the development of ulcers in CD patients is the increased immunological activity of the epithelium.^[16]^ Several studies examining saliva in patients with IBD have found elevated levels of proinflammatory cytokines compared with controls. Regarding oral lesions and salivary proinflammatory cytokine levels, it was found that elevated salivary interleukin (IL)-6 and TNF-α levels correlate with oral lesions. The bacteria involved in oral ulcers stimulate host oral cells to produce various cytokines such as IL-8 and TNF-α, further aggravating tissue destruction in this disease. Thus, the oral diseases in IBD might be related to overexpressed proinflammatory cytokine levels in the oral cavity of IBD patients due to the altered systemic immune balance.^[20]^ Specifically, bacteria involved in oral ulcers stimulate oral cells to produce various cytokines, such as IL-6 and TNF-α, which further exacerbate tissue damage in this disease. Therefore, oral diseases in IBD may be linked to elevated levels of proinflammatory cytokines in the oral cavity, resulting from an altered systemic immune response.^[20]^

Medical therapy for these oral lesions can be difficult, and there is little research available on how well medical treatment works for oral CD lesions. Considering this case report, the patient experienced severe major aphthous ulcers accompanied by extreme pain that interfered with his daily life. In addition, treatment includes topical steroids, and systemic steroids may be administered in cases of more severe presentations.^[21]^ The treatment in our case was combined depending on the severity of the condition seen.

First strategy: Triamcinolone acetonide is a medium-to-high-potency corticosteroid, is a fluorinated prednisolone derivative, and considered an intermediate-acting glucocorticoid.^[22]^ He also works as an anti-inflammatory, analgesic, and antiallergic, properties that make it a useful treatment for aphthous ulcers. Each of their actions delays stomatitis and promotes ulcers healing. Triamcinolone acetonide has been demonstrated to suppress fibroblast proliferation and collagen formation. TGF-β expression has been shown to decrease when fibroblasts are treated with triamcinolone acetonide.^[23]^ It is safe and effective in a randomized control trial conducted on adults.^[21]^ To prevent systemic adverse effects, corticosteroids are typically used topically as ointments, pastes, lozenges, and mouthwashes. Nevertheless, ointments frequently leave the oral cavity when speaking, eating, or by salivation, as well as when swallowing and moving the tongue.^[24]^ That’s why it was used. Triamcinolone acetonide in the form of orabase, because of the mucoadhesive polymer in the formulations, could provide a longer contact time.^[25]^ On the other hand, continuous use of these medications increases the possibility of developing oral candidiasis. Moreover, the majority of pharmaceuticals may have negative side effects or additional disadvantages, which makes their clinical usage questionable. Consequently, doctors and dentists have been searching constantly for therapeutic approaches that can effectively manage aphthous ulcer symptoms without any adverse reactions.^[26]^ That’s why it was the second strategy: photobiomodulation by low-level laser. This therapy has broad scientific support in clinical and laboratory studies.^[27]^ PBMT was discovered by Ender Mester in the 1960s, and soon after, it was immediately apparent that PBMT had the miraculous ability to treat a wide range of diseases and conditions involving inflammation, pain, wound healing, regeneration, and abnormal immunological responses.^[28]^ Although the mechanisms underlying PBMT are complex, they basically depend on photoreceptors in specific subcellular components-specifically, the electron transport chain in the mitochondrial membrane absorbing visible red and near-infrared wavelengths.^[29]^ The axonal conduction velocity was slowed, and the action potential amplitude was reduced, indicating the inhibition of electrophysiological activity. Blocking these nerves hence results in less noxious impulses being transmitted, which lessens pain.^[30]^ Rocca et al^[31]^ discovered that the diode 635 nm was the device that had the earliest impact on lowering pain during the course of the treatment, comparable to those achieved with lasers that were 808 nm and 450 nm. The main benefit of PBMT is that it instantly reduces pain more than its effects on the number of cells in the repair; this is according to a study by Bayat et al.^[32]^ Consequently, we employed the combination of triamcinolone acetonide and PBMT to shorten the response time of treatment. Based on scientific evidence, a combination of corticosteroids and PBMT is able to heal major ulcers without any scars^[33]^ and mucositis.^[34]^ By working together, dentists and gastroenterologists can provide complete care and make sure treatment is appropriate and effective for patients. Dentists in particular struggle to manage painful mouth ulcers that negatively impact QoL.

This report is significant as it presents the first documented severe case of major aphthous ulcer caused by CD, along with the use of a combined treatment involving Triamcinolone acetonide and PBMT.

## Author contributions

**Data curation:** Shahed Kuraitby.

**Writing – original draft:** Shahed Kuraitby.

**Writing – review & editing:** Shahed Kuraitby.

**Supervision:** Abeer A. Aljoujou.
